# Gender differences in illness acceptance and coping strategies among patients with rheumatoid arthritis, psoriatic arthritis, and axial spondyloarthritis: a cross-sectional survey study

**DOI:** 10.1007/s00296-025-05805-7

**Published:** 2025-02-15

**Authors:** Luise Holberg Lindgren, Nanna Maria Hammer, Caroline A. Flurey, Kim Vilbæk Jensen, Lena Andersen, Bente Appel Esbensen

**Affiliations:** 1 Copenhagen Center for Arthritis Research, Center for Rheumatology and Spine Diseases, Centre for Head and Orthopaedics, Rigshospitalet, Valdemar Hansens Vej 17, Entrance 5, Glostrup, 2600 Denmark; 2 Department of Public Health, Faculty of Health and Medical Sciences, University of Copenhagen, Copenhagen, Denmark; 3 Universtiy of the West of England, Bristol, UK; 4 Patient Research Partner, Copenhagen, Denmark; 5 Department of Clinical Medicine, Faculty of Health and Medical Sciences, University of Copenhagen, Copenhagen, Denmark

**Keywords:** Arthritis, Male, Female, Cross-sectional studies, Surveys and questionnaires, Illness acceptance, Coping, Patient-reported outcome measures

## Abstract

This study aimed to investigate potential gender differences in illness acceptance and coping strategies in patients with inflammatory arthritis (IA). Furthermore, the study aimed to identify factors associated with illness acceptance and coping strategies specific to men and women. A cross-sectional nationwide survey design was applied. Illness acceptance was measured by using the Acceptance of Illness Scale and coping was measured by using the Medical Coping Modes Questionnaire. Descriptive statistics were used to explore gender differences in illness acceptance and coping strategies in patients with IA, while logistic regression analyses investigated associated factors. The study included 664 participants (85.1% women) with a mean age of 50 and median disease duration of 10 years. Diagnoses included 53.3% rheumatoid arthritis, 27.1% psoriatic arthritis, and 19.6% axial spondyloarthritis. A statistically significant difference was found between men and women in use of avoidance (*P* = 0.015). Higher illness acceptance was associated with tertiary education in men (OR: 3.90) and older age in women (OR: 1.35 per 10 years). Women with higher disease activity used confrontation more (OR: 1.64) than women with less activity. Men relied more on avoidance when facing psychological distress (OR: 1.29) or severe fatigue (OR: 1.31), as did women with high disease activity (OR: 2.09). Acceptance-resignation was linked to higher disability and psychological distress in men (OR: 1.32 and 1.52) and higher disease activity in women (OR: 2.09). We identified factors associated with illness acceptance and coping strategies among IA patients. Gender-sensitive approaches are needed to address specific factors influencing illness acceptance and coping in men and women.

## Introduction

Inflammatory arthritis (IA), comprising rheumatoid arthritis (RA), psoriatic arthritis (PsA) and axial spondylarthritis (axSpA), encompasses a spectrum of acute and chronic joint conditions marked by symptoms such as joint pain, swelling, and tenderness, stemming from underlying inflammation [1–3]. Patients living with IA experience unpredictable symptom fluctuations, functional limitations, diminished participation in daily activities, and the ensuing emotional consequences [4, 5]. As a result, patients must manage the effects of fluctuating symptoms and treatments on their daily lives while coping with the emotional toll of their illness [6]. Active involvement in self-management empowers patients to reduce the impact of IA and accept their illness - a process that has been shown to significantly improve emotional well-being and lower levels of anxiety and depression [7–9]. Previous research suggests that coping strategy development varies by gender [10]. Women tend to adapt their coping strategies more readily, often using problem-solving and emotion-focused techniques more frequently than men [11]. Additionally, women are more likely to benefit from stronger support networks and social reinforcement, which can enhance their coping abilities [12]. In contrast, men may face greater challenges in accepting their illness, potentially due to societal norms related to masculinity [13]. In addition, evidence suggests that men may require more time to acclimate and reconcile with their condition compared to women [8, 13]. This delay in acceptance could exacerbate psychological distress and hinder effective coping strategies living with IA.

Understanding whether men and women with IA differ in their coping strategies and if so, what these differences are, can enable tailored support provision. Gender does not exist in isolation, therefore by identifying the demographic and clinical factors linked to coping strategies and acceptance in both genders, healthcare professionals (HPs) can tailor interventions to target more specific groups of men and women. Coping and acceptance have previously been investigated in patients with rheumatic disease, but to date few studies have aimed to define factors associated with illness acceptance and coping strategies in men and women [13, 14].

Consequently, the aim of this study was twofold: first, to investigate the presence of gender differences in illness acceptance and coping strategies in patients with IA and second, to identify the demographic and clinical factors associated with illness acceptance and coping strategies specific to men and women, respectively.

## Methods

### Study design and data collection

The study was conducted as a cross-sectional survey. Data were collected by a questionnaire set up in the online survey program SurveyXact (© 2013–2015 Rambøll). The study adhered to the latest recommendations for reporting survey studies [15].

### Participants and setting

Eligible participants were ≥ 18 years old and diagnosed with RA, PsA or axSpA (including ankylosing spondylitis (AS)). Participants were recruited from March through May 2016. Invitation material about the study was shared with a link and QR code, distributed through the Danish Rheumatism Association (newsletter, Facebook group, and members magazine), local arthritis networks/groups (email), diagnosis networks (Facebook groups), and across the country on posters and leaflets in the hospitals’ rheumatology departments. There were no exclusion criteria in this study.

### Outcomes and instruments

Socio-demographics, diagnosis, clinical characteristics, disease activity, functional status, symptoms, coping and illness acceptance were obtained through a self-reported online questionnaire.

*Socio-demographics.* The socio-demographic factors assessed included gender, age, marital status, education, and occupational status.

*Clinical characteristics.* Clinical characteristics included self-reported diagnosis with RA, PsA or axSpA, disease duration in years, current treatment with disease modifying anti-rheumatic drugs (DMARD), and the presence of co-morbidities.

*Disease activity.* For RA, disease activity was measured using the 3 item Patient-based Disease Activity Score 2 (PDAS2) [16] and for axSpA, disease activity was measured using the 6 item Bath Ankylosing Spondylitis Disease Activity Index (BASDAI) [17]. Participants with PsA answered the questions of both PDAS2 and BASDAI, as PsA can manifest as both peripheral and axial disease.

*Symptoms.* Arthritis-related symptoms - pain and patient global assessment (i.e., evaluation of the overall impact of arthritis on everyday life)– were measured using 0-100 visual analog scale (VAS). Fatigue was measured by the Bristol Rheumatoid Arthritis Fatigue– Numerical Rating Scales (BRAF-NRS) [18] consisting of three items measuring (1) severity, (2) effect, and (3) coping.

*Functional status.* Physical functional status was measured by the Health Assessment Questionnaire (HAQ) Disability Index including two questions on walking and sports from the multidimensional health assessment questionnaire MDHAQ [19]. In addition, psychological status was measured by using the three psychological questions from MDHAQ (sleep, anxiety and depression) [19].

*Illness acceptance.* Participants’ acceptance of their disease was measured using the Acceptance of Illness Scale (AIS) [20], which consists of eight items rated on a five-point Likert scale, and assesses participants’ acceptance of their condition, ability to feel valuable despite their condition, dependency, and feelings of ‘uselessness’. The total, a score of between 8 and 40 points, provides a measure of illness acceptance. Scores below 20 points are considered low and indicate no or poor acceptance and adaptation to illness. Scores between 20 and 30 points indicate a moderate level of acceptance, whereas scores above 30 points indicate high or full acceptance of one’s condition [20]. The AIS was developed in patients with hypertension, diabetes mellitus, RA & systemic blood cancers.

*Coping.* Coping was evaluated using the Medical Coping Modes Questionnaire (MCMQ) [21]. The instrument consists of 19 items and assesses three types of coping strategies: (1) confrontation: covering attempts to meet the illness, primarily through information gathering and active involvement in treatment (eight items); (2) avoidance: covering attempts to escape thinking about the illness through distraction, attention re-deployment, and processing less information (seven items); (3) acceptance-resignation: covering restriction in affective expression and more negative in expressed feeling (four items). As the term “acceptance-resignation” implies, this coping strategy refers to a passive way of coping with illness. It means a person feels resigned or defeated by the illness, rather than truly accepting it in a positive way. Each item uses a 4-point Likert scale and higher total scores on each scale indicate a greater use of that specific coping strategy. The MCMQ was developed among patients with life-threatening and non-life threatening conditions, including RA [21], to acknowledge the multifaceted nature of coping, and is based on the theory that there are three modes of responding to medical illness: tackling, avoiding and capitulating (passivity).

Additional outcomes As part of the survey, data on the participants’ preferences for self-management and support services were collected and are reported elsewhere [22].

The survey was pretested and reviewed by our patient research partners.

### Statistical analyses

Data are presented as means with standard deviations (SD), medians with interquartile ranges (IQR) and frequencies where appropriate. Between-group differences according to gender were tested using independent t-test/Mann-Whitney U test for continuous data and χ^2^/Fisher’s exact test for categorical data with the significance value set at *p* < 0.05 (two-tailed).

To test for factors associated with illness acceptance and coping in men and in women, logistic regression analyses were performed. Illness acceptance (i.e., AIS) and the three different coping strategies (i.e., MCMQ confrontation, avoidance, and acceptance-resignation) were tested as dependent variables in the analyses. All logistic regression analyses were gender specific and thus were run separately for men and women, respectively.

For the regression analyses, data on the dependent variables were dichotomized according to the median score on each scale (i.e., AIS > 25, MCMQ confrontation > 18, MCMQ avoidance > 17, and MCMQ acceptance-resignation > 9). Furthermore, data on disease activity were dichotomized according to the median scores on the PDAS and BASDAI scales. Accordingly, high disease activity was defined as a score above the median score in PDAS (> 4.2) for participants with RA, a score above the median score in the BASDAI (> 5.8) for participants with axSpA, and a score above the median score in either PDAS and/or BASDAI for participants with PsA. All included variables were tested in univariate logistic regression analyses. Considering the risk of finding significant results due to multiple testing, we only reported on highly significant results defined as *p* < 0.01 in the regression analyses [23]. Variables significant at a *p* < 0.1-level were included in multivariable analyses using backward selection. All analyses were performed using IBM Statistics SPSS v. 22.

### Patient research partners

As recommended by the European Alliance of Associations for Rheumatology (EULAR) [24] two patients (KVJ & LA; male and female, respectively), both diagnosed with RA, were included as equal research partners in all relevant phases of the study.

## Results

In total, 664 participants were included in the study, with 85.1% being women (Table 1). They were well-represented from across the entire country. The mean age was 50, and men were significantly older than women (*p* = 0.014). Additionally, 74.6% of participants were in a relationship (married or partnered), with significantly more men in a relationship (83.9%; *p* = 0.005). Overall, 50.4% were working, with comparable proportions of men and women employed (full-time or part-time). However, more men were working full-time (*p* = 0.001), and significantly more women had higher education (*p* = 0.013). The median disease duration was 10 years. Disease distribution showed 53.3% had RA, 27.1% had PsA, and 19.6% had axSpA, with significantly more women diagnosed with RA and more men with axSpA (*p* < 0.001). No significant differences between women and men were found in physical and psychological status or in symptoms (pain, global impact, and fatigue). There were no significant differences between women and men in illness acceptance, with a median score of 25 (IQR 20.0–31.0) for the entire population. Regarding coping strategies, men used avoidance significantly less (*p* = 0.015). There were no significant gender differences in the active coping strategy of confrontation or the passive acceptance-resignation strategy.

**Table 1:** Characteristics of the study population according to gender

Variables	All (*N* = 664)	Men (*n* = 99)	Women (*n* = 565)	*p*
**Socio-demographics**				
Age (years), mean (SD)	50.0 (12.9)	52.9 (12.4)	49.5 (12.9)	**0.014**
Marital status, (*n* = 662) *, n (%) Married/civil partnership Partner, living together Partner, not living together Single Divorced Widowed	365 (55.1) 100 (15.1) 29 (4.4) 111 (16.8) 40 (6.0) 17 (2.6)	67 (67.7) 15 (15.2) 1 (1.0) 14 (14.1) 0 2 (2.0)	298 (52.9) 85 (15.1) 28 (5.0) 97 (17.2) 40 (7.1) 15 (2.7)	**0.005**
Education, (*n* = 660) *, n (%) Lower secondary Higher secondary Tertiary	58 (8.8) 289 (43.8) 313 (47.4)	14 (14.1) 50 (50.5) 35 (35.4)	44 (7.8) 239 (42.6) 278 (49.6)	**0.013**
Occupation, (*n* = 656) *, n (%) Full-time work Part-time work Unemployed, due to arthritis Unemployed, for other/unknown reasons Retired Student	176 (26.8) 155 (23.6) 95 (14.5) 68 (10.4) 132 (20.1) 30 (4.6)	37 (37.4) 10 (10.1) 17 (17.2) 9 (9.1) 25 (25.3) 1 (1.0)	139 (25.0) 145 (26.0) 78 (14.0) 59 (10.6) 107 (19.2) 29 (5.2)	**0.001**
**Disease and medications**				
Diagnosis, n (%) Rheumatoid arthritis (RA) Psoriatic arthritis (PsA) Axial spondyloarthritis (axSpA)	354 (53.3) 180 (27.1) 130 (19.6)	30 (30.3) 31 (31.3) 38 (38.4)	324 (57.3) 149 (26.4) 92 (16.3)	**< 0.001**
Disease duration (years) (*n* = 663) *, Median (IQR)	10.0 (4.0–16.0)	10.0 (5.0–24.0)	9.5 (4.0–16.0)	0.135
Biological DMARD (yes), n (%)	211 (31.8)	40 (40.4)	171 (30.3)	**0.048**
Comorbidities (any), n (%)	315 (47.4)	42 (42.2)	273 (48.3)	0.326
Disease activity				
PDAS (2,7–7,9) (*RA + PsA, n* = 535), Median (IQR)^#^	4.2 (3.5-5.0)	4.1 (3.3–5.2)	4.2 (3.5-5.0)	0.760
BASDAI (0–10) (*axSpA + PsA*, *n* = 310), Median (IQR)^#^	5.8 (4.0-7.1)	5.8 (3.3-7.0)	5.8 (4.2–7.2)	0.295
Functional status				
Physical (MD-HAQ) (0–10), Median (IQR)^#^	2.3 (1.0–4.0)	2.3 (0.7-4.0)	2.3 (1.0–4.0)	0.571
Psychological (MD-HAQ) (0-9.9), Median (IQR)^#^	3.3 (1.1–4.4)	3.3 (1.1–4.4)	3.3 (1.1–4.4)	0.969
**Symptoms**				
Pain (VAS) (0-100), Median (IQR)^#^	50.0 (23.0–70.0)	51.0 (18.0–70.0)	50.0 (24.0–70.0)	0.802
Global (VAS) (0-100), Median (IQR)^#^	55.5 (28.0–75.0)	58.0 (25.0–75.0)	55.0 (28.0–75.0)	0.918
Fatigue, severity (BRAF-NRS) (0–10), Median (IQR)^#^	7.0 (5.0–8.0)	7.0 (5.0–8.0)	7.0 (5.0–8.0)	0.163
Fatigue, effect (BRAF-NRS) (0–10), Median (IQR)^#^	7.0 (4.0–8.0)	7.0 (4.0–7.0)	7.0 (4.0–8.0)	0.127
Fatigue, cope (BRAF-NRS) (0–10), Median (IQR)^#^	6.0 (4.0–8.0)	6.0 (4.0–8.0)	6.0 (4.0–8.0)	0.925
**Illness acceptance and coping strategies**				
Illness acceptance (AIS total score) (8–40), Median (IQR)^#^	25.0 (20.0–31.0)	26.0 (20.0–31.0)	25.0 (20.0–30.0)	0.378
Confrontation (MCMQ) (8–32), Median (IQR)^#^	18.0 (16.0–20.0)	18.0 (17.0–20.0)	18.0 (16.0–20.0)	0.294
Avoidance (MCMQ) (7–28), Median (IQR)^#^	17.0 (15.0–19.0)	16.0 (13.0–18.0)	17.0 (15.0–19.0)	**0.015**
Acceptance-resignation (MCMQ) (4–16), Median (IQR)^#^	9.0 (7.0–10.0)	9.0 (7.0–10.0)	9.0 (7.0–10.0)	0.650

p: Tests of differences between male and female participants (independent T-test/Mann-Whitney U Test for continuous data and χ^2^/Fisher’s Exact Test for categorical data); SD: Standard deviation; IQR: Inter quartile range; DMARD: Disease modifying anti-rheumatic drugs; PDAS: Patient-based Disease Activity Score; BASDAI: Bath Ankylosing Spondylitis Disease Activity Index; MD-HAQ: Multi-Dimensional Health Assessment Questionnaire; VAS: Visual analogue scale; BRAF-NRS: Bristol Rheumatoid Arthritis Fatigue– Numerical Rating Scale; AIS: Acceptance of Illness Scale; MCMQ: Medical Coping Modes Questionnaire

*: *n* < 664 due to missing data; #: A higher score indicates higher levels of disease activity (PDAS and BASDAI), higher levels of functional disability (MD-HAQ physical) and psychological distress (MD-HAQ psychological), higher levels of pain (VAS pain) and perceived overall impact of disease (VAS global), higher levels of fatigue severity (BRAF-NRS severity ) and fatigue’s effect on life (BRAF-NRS effect), and fewer problems in coping with fatigue (BRAF-NRS coping), a higher level of illness acceptance (AIS) score and indicates increased use of the different coping strategies (MCMQ confrontation, avoidance and acceptance-resignation). Significant results (*p* < 0.05) are in **bold**

### Factors associated with illness acceptance

For men, tertiary education (OR: 3.90; CI = 1.60–9.94) was significantly associated with a high degree of illness acceptance (Table 2). In women, an increase in age (OR: 1.35; CI = 1.18–1.54 per 10-year increase) was significantly associated with a high degree of illness acceptance.

**Table 2:** Logistic regression analyses of factors associated with **high degree of illness acceptance.** (complete case analyses)

	Men (*n* = 99)		Women (*n* = 557–565)	
	OR (95% CI)	p	OR (95% CI)	p
Age, per 10-year increase	1.15 (0.83–1.59)	0.393	**1.35 (1.18–1.54)**	**< 0.001**
Marital status (if single)	0.38 (0.12–1.20)	0.100	1.35 (0.93–1.96)	0.119
Education (if tertiary)	**3.90 (1.60–9.49)**	**0.003**	1.48 (1.06–2.07)	0.021
Occupation (if employed)	2.18 (0.98–4.87)	0.057	1.08 (0.77–1.50)	0.674
Diagnosis (RA as reference) Psoriatic arthritis Axial spondyloarthritis	0.55 (0.20–1.52) 0.60 (0.23–1.58)	0.456 0.248 0.302	0.70 (0.48–1.04) 0.68 (0.43–1.08)	0.101 0.076 0.105
Disease duration, per 1-year increase	1.00 (0.98–1.03)	0.797	1.02 (1.00-1.03)	0.044
Current biological treatment (if yes)	0.49 (0.22–1.11)	0.087	0.81 (0.57–1.17)	0.261
Comorbidities (if any)	2.66 (1.17–6.06)	0.020	0.81 (0.58–1.13)	0.218
Disease activity (if high) *	**0.33 (0.14–0.74)**	**0.007**	**0.38 (0.27–0.54)**	**< 0.001**
Physical (MD-HAQ) (0–10), per 1-unit increase^#^	**0.59 (0.46–0.76)**	**< 0.001**	**0.69 (0.62–0.76)**	**< 0.001**
Psychological (MD-HAQ) (0-9.99), per 1-unit increase^#^	**0.66 (0.54–0.81)**	**< 0.001**	**0.72 (0.66–0.78)**	**< 0.001**
Pain (VAS) (0–10), per cm. increase^#^	**0.78 (0.67–0.90)**	**0.001**	**0.77 (0.72–0.83)**	**< 0.001**
Global (VAS) (0–10), per cm. increase^#^	**0.74 (0.63–0.86)**	**< 0.001**	**0.74 (0.69–0.80)**	**< 0.001**
Fatigue; severity (BRAF-NRS) (0–10), per 1-unit increase^#^	**0.76 (0.63–0.91)**	**0.004**	**0.76 (0.70–0.82)**	**< 0.001**
Fatigue, effect BRAF-NRS) (0–10), per 1-unit increase^#^	**0.74 (0.62–0.89)**	**0.001**	**0.77 (0.71–0.82)**	**< 0.001**
Fatigue, coping BRAF-NRS) (0–10), per 1-unit increase^#^	**1.25 (1.06–1.47)**	**0.007**	**1.19 (1.11–1.28)**	**< 0.001**

Univariate regression analyses of factors associated with high degree of illness acceptance defined as a score above the median (> 25) on the Acceptance of Illness Scale (AIS) in men and women, respectively. OR: Odds ratio; CI: Confidence interval; RA: Rheumatoid arthritis; MD-HAQ: Multi-Dimensional Health Assessment Questionnaire; VAS: Visual analogue scale; BRAF-NRS: Bristol Rheumatoid Arthritis Fatigue– Numerical Rating Scales

*: High disease activity is defined as a score above the median score (> 4.2) in the Patient-based Disease Activity Score (PDAS) for participants with RA, a score above the median score (> 5.8) in the Bath Ankylosing Spondylitis Disease Activity Index (BASDAI) for participants with axSpA, and a score above the median score in either PDAS and/or BASDAI for participants with PsA; #: A higher score indicates higher levels of functional disability (MD-HAQ physical) and psychological distress (MD-HAQ psychological), higher levels of pain (VAS pain) and perceived overall impact of disease (VAS global), higher levels of fatigue severity (BRAF-NRS severity ) and fatigue’s effect on life (BRAF-NRS effect), and fewer problems in coping with fatigue (BRAF-NRS coping ). Highly significant results (*p* < 0.01) are **bold**

In both genders, an increase in disease activity, physical disability and emotional distress, pain, VAS-global, severity of fatigue, and effect of fatigue were significantly negativity associated with a high degree of illness acceptance. Furthermore, a better ability to cope with fatigue increased the odds of a high degree of illness acceptance in both genders.

The multivariable analysis (Table 3) showed that less physical disability remained significantly independently associated with a high degree of illness acceptance in the multivariable analysis in both men (OR: 0.56; CI = 0.42–0.75) and women (OR: 0.80; CI = 0.70–0.91).

**Table 3:** Multivariable logistic regression analyses of factors associated with **high degree of illness acceptance**. (complete case analyses)

	Men (*n* = 99)		Women (*n* = 557–565)	
	OR (95% CI)	p	OR (95% CI)	p
Age, per 10-year increase			**1.28 (1.10–1.49)**	**0.002**
Marital status (if single)				
Education (if tertiary)	3.05 (1.07–8.66)	0.036		
Occupation (if employed)				
Diagnosis (RA as reference) Psoriatic arthritis Axial spondyloarthritis				
Disease duration, per 1-year increase			1.02 (1.00-1.04)	0.035
Current biological treatment (if yes)				
Comorbidities (if any)	3.31 (1.19–9.26)	0.022		
Disease activity (if high) *				
Physical (MD-HAQ) (0–10), per 1-unit increase^#^	**0.56 (0.42–0.75)**	**< 0.001**	**0.80 (0.70–0.91)**	**0.001**
Psychological (MD-HAQ) (0-9.99), per 1-unit increase^#^			**0.86 (0.77–0.95)**	**0.002**
Pain (VAS) (0–10), per cm. increase^#^	**†**		**†**	
Global (VAS) (0–10), per cm. increase^#^			**0.87 (0.79–0.96)**	**0.004**
Fatigue, severity (BRAF-NRS) (0–10), per 1-unit increase^#^				
Fatigue, effect (BRAF-NRS) (0–10), per 1-unit increase^#^	**††**		**††**	
Fatigue, coping (BRAF-NRS) (0–10), per 1-unit increase^#^			**1.15 (1.06–1.25)**	**0.001**

Multivariable logistic regression analyses of factors associated with high degree of illness acceptance defined as a score above the median (> 25) on the Acceptance of Illness Scale (AIS) in men and women, respectively. OR: Odds ratio; CI: Confidence interval; RA: Rheumatoid arthritis; MD-HAQ: Multi-Dimensional Health Assessment Questionnaire; VAS: Visual analogue scale; BRAF-NRS: Bristol Rheumatoid Arthritis Fatigue– Numerical Rating Scales

*: High disease activity is defined as a score above the median score (> 4.2) in the Patient-based Disease Activity Score (PDAS) for participants with RA, a score above the median score (> 5.8) in the Bath Ankylosing Spondylitis Disease Activity Index (BASDAI) for participants with axSpA, and a score above the median score in either PDAS and/or BASDAI for participants with PsA; #: A higher score indicates higher levels of functional disability (MD-HAQ physical) and psychological distress (MD-HAQ psychological), higher levels of pain (VAS pain) and perceived overall impact of disease (VAS global), higher levels of fatigue severity (BRAF-NRS severity ) and fatigue’s effect on life (BRAF-NRS effect), and fewer problems in coping with fatigue (BRAF-NRS coping ). †: Variable not included in multivariable analysis due to strong correlation (> 0.7) with VAS global; ††: Variable not included in multivariable analysis due to strong correlation (> 0.7) with BRAF-NRS severity. Highly significant results (*p* < 0.01) are **bold**

### Factors associated with coping strategies

#### Confrontation as a coping strategy (Table 4)

**Table 4:** Logistic regression analyses of factors associated with the use of **coping strategies**. (complete case analyses)

	Confrontation as a coping strategy				Avoidance as a coping strategy				Acceptance-resignation as a coping strategy			
	Men (*n* = 99)		Women (*n* = 557–565)		Men (*n* = 99)		Women (*n* = 557–565)		Men (*n* = 99)		Women (*n* = 557–565)	
	OR (95% CI)	p	OR (95% CI)	p	OR (95% CI)	p	OR (95% CI)	p	OR (95% CI)	p	OR (95% CI)	p
Age, per 10-year increase	1.12 (0.81–1.55)	0.480	0.91 (0.80–1.03)	0.139	1.02 (0.72–1.44)	0.913	0.91 (0.80–1.04)	0.176	0.73 (0.52–1.03)	0.077	0.91 (0.80–1.04)	0.176
Marital status (if single)	0.28 (0.08–0.95)	0.040	0.73 (0.50–1.06)	0.100	1.00 (0.31–3.16)	0.995	1.04 (0.71–1.52)	0.849	1.86 (0.63–5.48)	0.259	1.04 (0.71–1.52)	0.849
Education (if tertiary)	0.66 (0.29–1.52)	0.330	0.88 (0.63–1.23)	0.452	0.82 (0.33–2.02)	0.664	1.33 (0.95–1.86)	0.102	1.72 (0.74–4.02)	0.206	1.33 (0.95–1.86)	0.102
Occupation (if employed)	0.89 (0.40–1.95)	0.761	0.76 (0.54–1.06)	0.104	0.99 (0.43–2.33)	0.990	0.80 (0.57–1.13)	0.210	0.85 (0.38–1.92)	0.696	0.80 (0.57–1.13)	0.210
Diagnosis (RA as reference) Psoriatic arthritis Axial spondyloarthritis	0.82 (0.30–2.25) 1.11 (0.43–2.90)	0.825 0.705 0.829	1.05 (0.71–1.55) 0.97 (0.61–1.55)	0.955 0.825 0.889	3.61 (1.09–11.94) 2.60 (0.81–8.39)	0.105 0.035 0.110	1.08 (0.72–1.60) 1.09 (0.68–1.75)	0.902 0.719 0.715	4.27 (1.37–13.32) 2.61 (0.86–7.89)	0.044 0.013 0.089	1.08 (0.72–1.60) 1.09 (0.68–1.75)	0.902 0.719 0.715
Disease duration, per 1-year increase	1.00 (0.97–1.03)	0.930	0.99 (0.97–1.01)	0.194	1.03 (1.00-1.06)	0.102	1.02 (1.00-1.03)	0.067	0.99 (0.96–1.02)	0.484	1.02 (1.00-1.03)	0.067
Current biological treatment (if yes)	1.45 (0.65–3.25)	0.368	1.26 (0.88–1.81)	0.206	1.33 (0.56–3.14)	0.515	1.17 (0.82–1.69)	0.390	1.01 (0.44–2.31)	0.983	1.17 (0.82–1.69)	0.390
Comorbidities (if any)	1.22 (0.55–2.72)	0.622	0.72 (0.52–1.01)	0.053	0.97 (0.41–2.30)	0.947	0.99 (0.71–1.39)	0.973	1.50 (0.66–3.42)	0.334	0.99 (0.71–1.39)	0.973
Disease activity (if high) *	1.84 (0.83–4.09)	0.133	**1.64 (1.18–2.30)**	**0.004**	3.00 (1.23–7.34)	0.016	**2.09 (1.48–2.94)**	**< 0.001**	2.73 (1.17–6.36)	0.020	**2.09 (1.48–2.94)**	**< 0.001**
Physical MD-HAQ (0–10), per 1-unit increase^#^	1.12 (0.92–1.37)	0.251	**1.14 (1.05–1.25)**	**0.003**	1.23 (0.99–1.52)	0.059	**1.19 (1.09–1.30)**	**< 0.001**	**1.32 (1.07–1.64)**	**0.010**	**1.19 (1.09–1.30)**	**< 0.001**
Psychological MD-HAQ (0-9.99), per 1-unit increase^#^	0.96 (0.82–1.12)	0.590	1.06 (0.98–1.14)	0.126	**1.29 (1.07–1.54)**	**0.005**	1.08 (1.00-1.16)	0.047	**1.52 (1.24–1.88)**	**< 0.001**	1.08 (1.00-1.16)	0.047
Pain VAS (0–10), per cm. increase^#^	1.11 (0.97–1.28)	0.119	1.08 (1.02–1.15)	0.015	1.19 (1.02–1.39)	0.025	**1.11 (1.04–1.19)**	**0.001**	1.16 (1.00-1.34)	0.044	**1.11 (1.04–1.19)**	**0.001**
Global VAS (0–10), per cm. increase^#^	1.10 (0.96–1.26)	0.178	1.07 (1.01–1.14)	0.031	1.14 (0.98–1.32)	0.095	**1.09 (1.02–1.16)**	**0.007**	**1.23 (1.06–1.43)**	**0.007**	**1.09 (1.02–1.16)**	**0.007**
Fatigue, severity (BRAF-NRS) (0–10), per 1-unit increase^#^	1.05 (0.89–1.24)	0.556	1.03 (0.96–1.10)	0.476	1.28 (1.04–1.59)	0.023	**1.15 (1.07–1.24)**	**< 0.001**	**1.51 (1.19–1.91)**	**0.001**	**1.15 (1.07–1.24)**	**< 0.001**
Fatigue, effect (BRAF-NRS) (0–10), per 1-unit increase^#^	1.02 (0.87–1.18)	0.851	1.00 (0.94–1.07)	0.918	**1.31 (1.07–1.59)**	**0.008**	**1.11 (1.04–1.18)**	**0.002**	**1.44 (1.17–1.78)**	**0.001**	**1.11 (1.04–1.18)**	**0.002**
Coping (BRAF-NRS) (0–10), per 1-unit increase^#^	1.10 (0.94–1.27)	0.231	0.99 (0.93–1.06)	0.747	0.90 (0.76–1.05)	0.177	0.98 (0.92–1.05)	0.544	0.86 (0.74–1.01)	0.060	0.98 (0.92–1.05)	0.544

Univariate regression analyses of factors associated with a greater use of coping strategies defined as a score above the median (> 18) of the sub scale in the Medical Coping Modes Questionnaire in men and women, respectively

OR: Odds ratio; CI: Confidence interval; RA: Rheumatoid arthritis; MD-HAQ: Multi-Dimensional Health Assessment Questionnaire; VAS: Visual analogue scale; BRAF-NRS: Bristol Rheumatoid Arthritis Fatigue– Numerical Rating Scales

*: High disease activity is defined as a score above the median score (> 4.2) in the Patient-based Disease Activity Score (PDAS) for participants with RA, a score above the median score (> 5.8) in the Bath Ankylosing Spondylitis Disease Activity Index (BASDAI) for participants with axSpA, and a score above the median score in either PDAS and/or BASDAI for participants with PsA; #: A higher score indicates higher levels of functional disability (MD-HAQ physical) and psychological distress (MD-HAQ psychological), higher levels of pain (VAS pain) and perceived overall impact of disease (VAS global), higher levels of fatigue severity (BRAF-NRS severity ) and fatigue’s effect on life (BRAF-NRS effect), and fewer problems in coping with fatigue (BRAF-NRS coping ). Highly significant results (*p* < 0.01) are **bold**

In men, no factors were found associated with the use of confrontation. For women, a higher degree of disease activity (OR = 1.64; CI = 1.18–2.30) and physical disability (OR = 1.14; CI = 1.05–1.25) was associated with more use of confrontation as a coping strategy. In the multivariable analysis, having comorbidities was associated with a reduced use of confrontation (OR = 0.63; CI = 0.45–0.89; Table 5).

**Table 5:** Multivariable logistic regression analyses of factors associated with the use of **coping strategies**. (complete case analyses)

	Confrontation as a coping strategy				Avoidance as a coping strategy				Acceptance-resignation as a coping strategy			
	Men (*n* = 99)		Women (*n* = 557–565)		Men (*n* = 99)		Women (*n* = 557–565)		Men (*n* = 99)		Women (*n* = 557–565)	
	OR (95% CI)	p	**OR (95% CI)**	p	OR (95% CI)	p	OR (95% CI)	p	OR (95% CI)	p	OR (95% CI)	p
Age, per 10-year increase											**0.74 (0.63–0.88)**	**< 0.001**
Marital status (if single)	0.28 (0.08–0.95)	0.040										
Education (if tertiary)												
Occupation (if employed)												
Diagnosis (RA as reference) Psoriatic arthritis Axial spondyloarthritis												
Disease duration, per 1-year increase												
Current biological treatment (if yes)												
Comorbidities (if any)			**0.63 (0.45–0.89)**	**0.009**								
Disease activity (if high) *							**1.73 (1.17–2.54)**	**0.006**				
Physical function (MD-HAQ) (0–10), per 1-unit increase^#^			**1.17 (1.07–1.28)**	**0.001**							1.16 (1.01–1.32)	0.030
Psychological function (MD-HAQ) (0-9.99), per 1-unit increase^#^					**1.29 (1.07–1.54)**	**0.007**			**1.52 (1.24–1.88)**	**< 0.001**	**1.30 (1.17–1.45)**	**< 0.001**
Pain, VAS (0–10), per cm. increase^#^			**†**		**†**		**†**		**†**		**†**	
Global, VAS (0–10), per cm. increase^#^											**1.23 (1.11–1.36)**	**< 0.001**
Fatigue, severity (BRAF-NRS) (0–10), per 1-unit increase^#^							1.09 (1.00-1.19)	0.049				
Fatigue, effect (BRAF-NRS) (0–10), per 1-unit increase^#^					**††**		**††**		**††**		**††**	
Fatigue, coping (BRAF-NRS) (0–10), per 1-unit increase^#^											0.90 (0.82–0.98)	0.018

Multivariable logistic regression analyses of factors associated with a greater use of coping strategies defined as a score above the median (> 18) of the sub scales in the Medical Coping Modes Questionnaire in men and women, respectively. OR: Odds ratio; CI: Confidence interval; RA: Rheumatoid arthritis; MD-HAQ: Multi-Dimensional Health Assessment Questionnaire; VAS: Visual analogue scale; BRAF-NRS: Bristol Rheumatoid Arthritis Fatigue– Numerical Rating Scales

*: High disease activity is defined as a score above the median score (> 4.2) in the Patient-based Disease Activity Score (PDAS) for participants with RA, a score above the median score (> 5.8) in the Bath Ankylosing Spondylitis Disease Activity Index (BASDAI) for participants with axSpA, and a score above the median score in either PDAS and/or BASDAI for participants with PsA; #: A higher score indicates higher levels of functional disability (MD-HAQ physical) and psychological distress (MD-HAQ psychological), higher levels of pain (VAS pain) and perceived overall impact of disease (VAS global), higher levels of fatigue severity (BRAF-NRS severity ) and fatigue’s effect on life (BRAF-NRS effect), and fewer problems in coping with fatigue (BRAF-NRS coping ). †: Variable not included in multivariable analysis due to strong correlation (> 0.7) with VAS global; ††: Variable not included in multivariable analysis due to strong correlation (> 0.7) with BRAF-NRS severity. Highly significant results (*p* < 0.01) are **bold**

#### Avoidance as a coping strategy (Table 4)

For men, a higher degree of psychological distress (OR = 1.29; CI = 1.07–1.54) and effect of fatigue OR = 1.31; CI = 1.07–1.59) was associated with the use of avoidance as a coping strategy. In women, high disease activity and increase in physical disability, pain, perceived global impact of disease (VAS-global), severity of fatigue and effect of fatigue was significantly associated with the use of avoidance as a coping strategy. The multivariable analysis (Table 5) indicated that men with a higher degree of psychological distress were more likely to use avoidance as a coping strategy (OR = 1.29; CI = 1.07–1.54). In women, a higher degree of disease activity (OR = 1.73; CI = 1.17–2.54) was associated with using avoidance as a coping strategy.

#### Acceptance-resignation as a coping strategy (Table 4)

For men, a higher degree of psychological distress (OR = 1.52; CI = 1.24–1.88) was positivity associated with the use of acceptance-resignation as a coping strategy. In women, higher disease activity (OR = 2.09; CI = 1.48–2.94) and pain (OR = 1.11; CI = 1.04–1.19) was associated with the use of acceptance-resignation as a coping strategy. In both men and women, a higher degree of physical disability, higher perceived global impact of disease (VAS Global), and higher severity and effect of fatigue was positivity associated with use acceptance-resignation as a coping strategy. The multivariable analysis (Table 5) indicated that both men and women with a higher degree of psychological distress had increased odds of employing acceptance resignation as a coping strategy (OR = 1.52 (CI: 1.24–1.88), and OR = 1.30 (CI: 1.17–1.45), respectively).

## Discussion

This study offers valuable insights into gender differences and factors associated with illness acceptance and coping strategies in men and women with IA. Although both genders exhibited similar levels of illness acceptance, they were influenced by distinct factors. Men’s acceptance was positively associated with higher education, suggesting that educational attainment may provide men with tools for managing their illness, and that men from lower educational attainment groups need more support. Acceptance among women increased with age, which may be attributed to the competing demands of family and work during younger years. Additionally, it suggests that life experience and maturity may aid in adapting to IA, and possible due to a perception that the condition poses less of a threat to their future, as found in previous research [25]. The median illness acceptance score for the overall sample was 25, reflecting a moderate level of acceptance [26], and consistent with previous findings among men with RA in the UK [27]. Given the median disease duration of 10 years, participants may have had time to adjust to their condition, characterizing them as “experienced patients” who have adapted to life with IA over time.

In coping strategies, an interesting and somewhat contradictory finding was that women with high levels of disease activity and significant physical disability tended to utilize all three coping strategies simultaneously. One might assume that employing confrontation as a coping strategy would diminish the reliance on acceptance and avoidance, as confrontation typically reflects a proactive approach. However, this was not observed in our results, which complicates their interpretation and applicability to clinical practice. This finding underscores that patients with IA face challenges on multiple fronts, and may need to employ various strategies concurrently to navigate their daily lives effectively. Confrontation is an active strategy and involves engagement through information gathering and treatment participation [28]. This approach is generally beneficial, promoting empowerment and a sense of control, which supports better health management outcomes [29, 30]. Although we did not find any factors highly significantly associated (i.e., *p* < 0.01) with the use of confrontation in men, there was a trend toward single men having a lower use of confrontation. Thus, men in relationships, might be more likely to adopt confrontation strategies, such as seeking social support and information related to their treatment. Studies indicate that men are generally less inclined to seek help compared to women, often delaying consultations with healthcare professionals [31]. Health behaviors among men are influenced by traditional masculine values emphasizing strength, independence, and self-reliance, which can discourage help-seeking for minor issues [32]. However, men with partners tend to seek medical help more frequently, often due to encouragement from their partners [33, 34]. This suggests a need in rheumatology clinical practice to focus on men who live alone, as they may be less likely to seek help or communicate their needs effectively.

Our findings indicated that men experiencing higher levels of psychological distress or severe fatigue were more likely to use avoidance as a coping strategy, while women with high disease activity, increased disability, pain, and fatigue also tended to avoid dealing with their illness. Though avoidance may offer short-term relief, it is generally counterproductive long-term, as it prevents individuals from effectively addressing their condition [10, 35]. Avoidance involves efforts to avoid thinking about the illness and is often seen as a less effective coping strategy, as it can lead to neglect of necessary care and worsen symptoms over time [35]. Comparing genders, we found that men used avoidance significantly less than women, aligning with prior studies suggesting that men more often employ active coping strategies like problem-solving [36]. However, socialization theory suggests that men might also deny or avoid problems to align with societal expectations to conceal emotions [37]. For men, a higher degree of psychological distress was associated with both avoidance and acceptance-resignation coping strategies. This supports previous research indicating that men with RA may socially withdraw when struggling to cope [27]. One qualitative study on men with early RA [38] identified avoidance as a coping strategy encompassing physical withdrawal from activities, like avoiding stairs or carrying groceries, and psychological withdrawal, such as refraining from discussing their illness and withdrawing from social engagements, especially due to fatigue. In the short term, avoidance may serve as a protective strategy to manage flare-ups or emotional distress. However, healthcare professionals should note that avoidant behaviors, like missing appointments, may signal underlying psychological distress, which has been associated with poorer outcomes [39].

Both men and women tended to rely on acceptance-resignation in response to greater physical disability, psychological distress, and fatigue, indicating a shared passive coping mode when the illness felt especially overwhelming. This underscores how symptom burden can push patients toward resigned acceptance. Acceptance-resignation, marked by limited emotional expression and negative feelings, reflects a sense of giving up rather than a proactive acceptance of the illness [21]. This passive approach can reduce quality of life and hinder progress in managing health outcomes [40].

In recent years, there has been an increasing focus on men’s health and on men being underrepresented in research [31]. The majority of research within IA has previously included patients with RA and, as a result, primarily women are included in available literature [41]. Therefore, our study is a significant contribution in that it provides insight into similarities and differences in illness acceptance and coping strategies in men and women with IA.

In our study, we identified key factors influencing illness acceptance and various coping strategies among our participants. It was evident that participants employed multiple coping modes and strategies depending on their circumstances, aligning with previous qualitative research which found that men with RA adapted their coping strategies based on the situation and the restrictions they experienced [38]. Consequently, it seems essential that healthcare professionals treat patients as whole individuals, considering their current disease state and personal coping strategies. Given the fluctuating nature of IA, which manifests differently over time, it is crucial to tailor interventions to an individual’s current needs. Special attention should be given to specific groups, such as younger women and men from lower educational backgrounds, who may require more targeted support. As suggested by de Ridder et al. [42], instead of solely focusing on why some individuals struggle to adjust to a chronic condition, it may be more beneficial to provide psychosocial interventions designed to assist those who find it challenging to adjust. A qualitative interview study with patients with RA [43] underscored that acceptance is not a linear process. Delayed diagnosis can hinder acceptance, leading to resistant behavior and difficulties in finding the optimal way to accept the condition [43]. Therefore, offering self-management interventions to patients with IA is essential for supporting them to adapt and achieve acceptance of their condition [44, 45]. Such offers may empower patients, improve their quality of life, and enhance their ability to manage their illness effectively [44, 45].

### Limitations and strengths

Firstly, this study is subject to potential selection bias, due to the self-selected nature of the population. It is well known that participants in surveys are more likely to be well-resourced [46], potentially with better ability to cope with and accept IA. Consequently, our findings may not be representative of the broader patient population.

Although we conducted a survey focusing on gender differences, only 15% of our participants were men. This is unsurprising in relation to the wider literature, which suggests that men are reluctant to engage in health surveillance [47]. They do not readily express themselves in relation to their health [48], and have a preference for ‘hard-science’ studies, such as drugs trials over qualitative or psychosocial research [49]. However, this leads to considerations about the kind of men who chose to answer the questionnaire. The questionnaire was primarily distributed through the Danish Rheumatism Association and thus primarily among those who had actively chosen membership. It is therefore reasonable to conclude that those men who answered were well-resourced and, specifically, attentive towards their arthritis. It is argued that men may be more likely than women to decline participation as ‘saying no’ signifies independence and control [50]. It is therefore possible that those men who chose not to take part could have been struggling to gain control in other areas of their life, which could be due to their condition. Higher response rates in men with RA have been seen where surveys specifically target male patients (e.g., a separately titled questionnaire for ‘men’s experiences/coping strategies’) [13]. Thus, future research should aim to target recruitment to ensurea representative sample of male patients. A final limitation to consider is the use of PDAS2 for measuring disease activity in RA and peripheral PsA. PDAS2 has not been validated for use in the PsA population. However, when combined with BASDAI, we consider it to be the most appropriate self-reported measure currently available for assessing disease activity in patients with PsA. Additionally, as a self-reported measure, PDAS2 carries the inherent risk of inaccuracies due to subjective reporting, which can result in either overestimation or underestimation of disease activity. Despite this limitation, it is important to highlight that PDAS2 is a validated tool for assessing patient-perceived disease activity and has been shown to provide reliable insights. A strength in our study is that we only used validated questionnaires developed specifically for patients with arthritis or patients with chronic conditions. It is also a strength that we included patient research partners from the very inception of the study idea, which strengthened the patient perspective throughout the entire study period. In addition, this study included a large number of patients with IA with national representation, including not only patients with RA, but also patients with axSpA and PsA– conditions that are often underrepresented in research. Consequently, this study is a significant contribution to the body of knowledge on axSpA and PsA.

## Conclusion

In conclusion, our results highlight distinct gender differences in illness and coping strategies among patients with IA. We found that women were more likely than men to use avoidance as a coping strategy, but no further gender differences were identified at a group level. Illness acceptance was higher in men with tertiary education and in older women. Disability, pain, and fatigue negatively impacted acceptance for both genders, while effective fatigue coping improved it. Men used confrontation less if single, while women used confrontation more with higher disease activity. Acceptance-resignation was associated with higher disability and disease impact in both genders. These findings highlight the need for gender-sensitive approaches in managing IA addressing the specific factors that influence illness acceptance and coping behaviors in men and women.

## Data Availability

BAE is willing to examine all requests for the full dataset during a period of 5 years from the date of this publication, in accordance with Danish legislation. BAE will be involved in any case of a query about access to data. Participants did not give consent for data sharing, but the presented data are anonymized, and risk of identification is low. No additional unpublished data are available.
